# A brief educational intervention to improve health service responsiveness to intimate partner violence: a mixed methods evaluation

**DOI:** 10.1186/s12913-026-14277-9

**Published:** 2026-03-11

**Authors:** Angela Tonge, Sumudu Avanthi Hewage, Sue Cumming, Catherine Walsh, Bridget Abell, David N. Borg, Brett Baxter, Rex Parsons, Tegwen Howell, Steven M. McPhail

**Affiliations:** 1 Social Work Department, Princess Alexandra Hospital, Brisbane, QLD Australia; 2 Australian Centre for Health Services Innovation, Centre for Healthcare Transformation, Queensland University of Technology (QUT), Brisbane, Queensland Australia; 3 Physiotherapy Department, Princess Alexandra Hospital, Brisbane, Queensland Australia; 4 Remote Resolve, Brisbane, Queensland Australia

**Keywords:** Intimate partner violence, Sensitive inquiry, Clinician training, Hospital intervention, Implementation research, Theoretical domains framework.

## Abstract

**Background:**

This study evaluated the feasibility and impact of a brief educational intervention on clinicians’ ability to identify and respond to intimate partner violence (IPV) in a large metropolitan hospital orthopaedics unit in Australia.

**Methods:**

A mixed methods design was used. The intervention for physiotherapists and social workers included a foundational session on Sensitive Inquiry theory and simulations, followed by two one-hour workshops. All 15 trained participants contributed qualitative data via pre- and post-training focus groups or interviews exploring IPV-related knowledge and perceptions. Data were inductively coded and mapped to the Theoretical Domains Framework. Quantitative analysis of de-identified hospital data compared IPV detection rates over 12 months pre- and post-training. Beta regression assessed IPV detection as the primary outcome, adjusting for gender, study week, and COVID-19 impact.

**Results:**

Qualitative analysis showed positive shifts across multiple domains in the Theoretical Domains Framework. Participants reported greater knowledge, confidence, and skills in IPV inquiry, alongside increased peer support and motivation to embed Sensitive Inquiry into routine care. Post-training reflections also highlighted emerging team-based optimism, intentional practice, and awareness of IPV’s broader impact on patient care. Quantitative analysis included 12,335 eligible patients (mean age 36.7 years; 38.6% female). Females had significantly higher odds of IPV-related encounters than males (OR = 3.5; 95% CI = 2.9, 4.1). However, no statistically significant change in IPV detection rates was observed.

**Conclusions:**

While the training improved clinicians’ attitudes and readiness to address IPV, this did not translate into increased detection. Future studies should explore the implementation of the Sensitive Inquiry approach complemented with regular training sessions as part of multi-faceted intervention strategies.

**Supplementary Information:**

The online version contains supplementary material available at 10.1186/s12913-026-14277-9.

## Background

The World Health Organization (WHO) defines intimate partner violence (IPV) as “any behaviour within an intimate relationship that causes physical, sexual, or psychological, harm” [1]. IPV is predominantly perpetrated by men against women and occurs across diverse cultural and socio-economic contexts [1–3]. The prevalence estimates of lifetime IPV range from 20% in the Western Pacific, 22% in high-income countries and Europe and 25% in the WHO Regions of the Americas to 33% in the WHO African region, 31% in the WHO Eastern Mediterranean Region, and 33% in the WHO South-East Asia region [4]. However, it is widely recognised that IPV is an underestimated problem [5–7].

Since the late 1990s, professional bodies have issued guidelines on identifying and responding to IPV [8–11] and the importance of responding to IPV has been recognised in national health plans and legislations [11–13]. In Queensland, Australia, the *Not Now*,* Not Ever* report [14] recommended incorporating IPV-related competencies into health professional accreditation. More recently, the National Plan to End Violence Against Women and Children 2022–2032 reaffirmed Australia’s commitment to ending IPV within a generation, highlighting the need to strengthen workforce capability and trauma-informed responses [11].

Despite growing efforts worldwide, health systems are more likely to overlook affected individuals than successfully identify and refer them to appropriate services [15–17]. This is likely attributable to several reasons. IPV is a complex social phenomenon, and affected individuals often seek medical attention only when absolutely necessary, rarely disclosing their experiences voluntarily [5, 18, 19]. In addition to these complexities, healthcare staff face barriers such as lack of privacy and time, unavailability of resources, limited institutional protocols, legal concerns as well as personal belief issues and attitudes that can impede care, including blaming victims or normalising IPV [20–23]. Such barriers result in missing opportunities for intervention, and prior work has highlighted the need for future research to identify healthcare interventions that women find beneficial, validate their experiences, enhance their sense of self and self-efficacy, and contribute to safer outcomes [18, 24].

Current interventions attempt to overcome some of these challenges by including training programs. Studies report short-term improvements in health professionals’ knowledge, confidence and enquiry about intimate partner and family violence following such training [24]. However, most of these training programs have not been explicitly grounded in survivor-informed Sensitive Inquiry principles, which can be used in sensitive practice to identify and provide care services for individuals affected by IPV [24, 25]. Embedding survivor-informed Sensitive Inquiry principles within IPV training is critical as knowledge acquisition alone often fails to achieve sustained practice change or safe enquiry. Gains in attitudes and confidence rarely translate into routine screening, appropriate responses to disclosure, or reduced risk of harm unless practitioners receive explicit guidance on trauma-informed questioning and referral [26]. It is also widely acknowledged that clinicians should not rely solely on patient disclosure of violence to provide appropriate healthcare [27, 28], making Sensitive Inquiry a suitable approach ahead of screening.

In the Australian state of Victoria, public hospitals have implemented the *Strengthening Hospital Responses to Family Violence* (SHRFV) training model, which embeds Sensitive Inquiry and sensitive practice principles across the health workforce [29]. In contrast, comparable structured training has not previously been available in Queensland public hospitals. This work aimed to examine whether the implementation of Sensitive Inquiry training developed based on the SHRFV training model could improve physiotherapist and social workers’ perceptions of their ability to engage in Sensitive Inquiry in a large Queensland public hospital orthopaedics setting, and whether this was associated with a change in the detection of IPV among those receiving care. Orthopaedic clinics are a common place of presentation for IPV victims, who often present with musculoskeletal injuries, making these clinics a key context in which health systems are expected to demonstrate responsiveness [17]. Healthcare staff in these clinics including physiotherapists and social workers are well positioned to identify signs of IPV, initiate timely referral, and coordinate appropriate support services. Their prolonged and close engagement with patients enables them to establish trust, which is foundational for conducting Sensitive Inquiry and facilitates embedding this approach into routine workflows. However, historically, physiotherapists have not received formal training in the identification and sensitive management of suspected IPV cases during their professional development [30–33].

To our knowledge, the current project represents the evaluation of the first training initiative in a Queensland public hospital for physiotherapists and social workers adopting Sensitive Inquiry principles to strengthen health service responses to IPV. We hypothesised that training physiotherapists and social workers in Sensitive Inquiry techniques would be feasible and would lead to higher rates of IPV detection among their care recipients, as well as the initiation of supportive interventions.

## Methods

### Study design, reporting and approvals

This study employed a mixed methods interrupted time-series design to address the two objectives. A mixed methods design was selected to enable the exploration of these potentially complex phenomena from multiple perspectives, combining the qualitative experiences of those who received the brief education intervention with quantitative healthcare system level data regarding detection rates. Clinicians’ perceptions of the training program were explored through a series of focus groups and semi-structured interviews with physiotherapists and social workers. An initial focus group was conducted to gather baseline information on participants’ knowledge, confidence, comfort, and current practice. This was followed by an educational session on IPV and Sensitive Inquiry. A subsequent focus group was then held post-training to assess changes in confidence, comfort, and clinical practice.

To determine whether the detection of individuals affected by IPV changed significantly after the training program, a quantitative analysis of routinely recorded clinical and service data was used. This comprised analysis of coded hospital data (ICD-10), over a 12-month period before and after the implementation of the brief Sensitive Inquiry training intervention. Both qualitative and quantitative data were considered equally important in contributing to the evaluation and analysed concurrently in a convergent parallel design.

The study is reported in accordance with the Good Reporting of A Mixed Methods Study (GRAMMS) checklist [34] as detailed in Supplementary Material Table S1. The TIDieR checklist [35] presented in Table S2 in the Supplementary Material was used to report the training program. Ethics approval was granted by Metro South Hospital and Health Service Human Research Ethics Committee (HREC), Australia (Ref No: HREC/19/QMS/57112).

### Study population and setting

The study was conducted with staff who work in a large orthopaedics unit of a major metropolitan hospital in Australia, which experiences a high demand for trauma-related orthopaedic services. The hospital serves a region that has consistently ranked fourth in the State for breaches of Domestic Violence Orders over the past decade [36]. Data collection was planned for a second regional site in Queensland but did not occur due to challenges associated with the COVID-19 pandemic response in that region.

### Participants for the quantitative component

Anonymised clinical data from routine hospital administrative records were extracted for one year prior and one year after the education intervention. This included male and female patients who were admitted to the study hospital and received one or more trauma-related clinical diagnoses based on the International Statistical Classification of Diseases and Related Health Problems, 10th Revision (ICD-10) codes. Patients were limited to those between 15 and 60 years of age, as the study was intended to focus on young people through to middle aged adults who frequent orthopaedic trauma units. Although detection of intimate partner and family violence affecting older adults is also an important topic worthy of investigation, it was not the focus of the present study.

The required sample size to detect a change in IPV detection rates was estimated a priori. Based on service activity data and historical IPV detection rates, we anticipated a minimum of 1,440 eligible cases involving women would contribute to the primary analysis, with at least 100 cases identified as IPV related. This sample size was estimated to provide > 80% power to detect an increase in IPV identification rates from 0.77% to 2.00% within the target population. Increasing the IPV detection rate to 2% was considered conservative, as a 2012 study reported that 16% of women presenting to the fracture clinics included in their study had experienced IPV within the past 12 months [17].

### Participants for the qualitative component

All orthopaedic physiotherapy and social work staff with potential involvement in the brief Sensitive Inquiry training at the study hospital (*n* = 15) were eligible and invited to participate in the qualitative component of the evaluation. All who completed the training (*n* = 15) consented to qualitative data collection, which was conducted before the first training session (pre-training) and immediately after the final session (post-training).

## Study procedures

### Training program

**Fig. 1:**
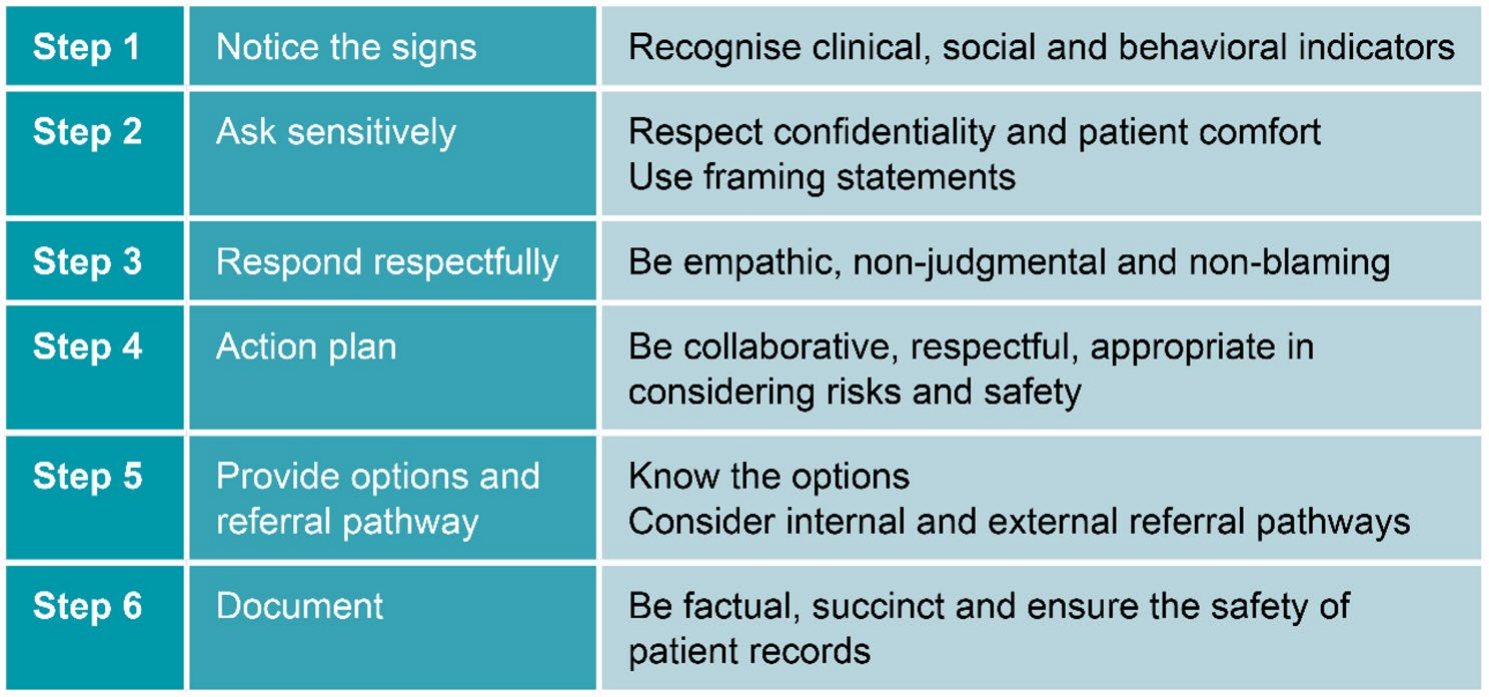
Six step sensitive inquiry model used in the training program

Based on the Sensitive Inquiry principles, the training program aimed to strengthen clinicians’ ability to recognize and respond to IPV by improving communication skills, confidence, comfort, and knowledge of referral pathways and documentation (Fig. 1). It consisted of two one-hour in-person workshops held during work hours, with two sessions scheduled to minimize workflow disruption. Although a larger multi-level implementation strategy was considered, organizational capacity constraints, including the COVID-19 response, necessitated evaluation of a brief, easily scalable educational intervention. The light-touch format also aligned with existing professional development practices at the study site and, if effective, would be pragmatic for wider implementation in similar healthcare settings.

The program was adapted from the *Strengthening Hospital Responses to Family Violence* service model, developed by the Royal Women’s Hospital and Bendigo Health, and widely implemented in Victorian hospitals [25, 29]. Grounded in the Sensitive Inquiry approach and aligned with the Multi-Agency Risk Assessment and Management Framework [37], *Strengthening Hospital Responses to Family Violence* service model draws on WHO’s case-finding principles and sensitive practice informed by survivor experiences [25].

The training program included practical tools such as training modules, case studies, role plays, and simulations to support skills development delivered by authors AT (Advanced Social Worker, Critical Care, with 30 years hospital experience) and CW (Advanced Social Worker. Domestic and Family Violence {DFV} Specialist, with 19 years hospital experience). A hospital-employed social work DFV specialist was responsible for implementing and monitoring the training program. Prior to completing the training, all eligible participants completed a foundational session covering essential theory and simulation exercises on Sensitive Inquiry. During the training, participants received practice guides and resources and engaged in reflective and peer-based learning.

Fidelity of the program was maintained by following a structured session format, including facilitator checklist for education content and activities, although contextual adjustments to the program were made based in response to the nature of participants and their organizational roles. Participants were given time to ask questions at the end of the workshop and were also provided with an email contact for follow-up queries. No additional remuneration was provided for participation, and time spent in training was within the context of their paid employment at the participating hospital.

### Quantitative data collection

The primary outcome measure for the quantitative component was the change in detection rates of individuals diagnosed with one or more trauma-related ICD-10 codes that can be indicative of IPV. This was assessed by using routinely recorded de-identified clinical and service data to compare the proportion of patients with relevant diagnoses per week, stratified by age and gender. Table S3 in the Supplementary Material presents the 20 most prevalent principal diagnoses during the study period. Detection rates were analysed for the 12-month period before and 12-month period after the implementation of the training program.

### Qualitative data collection

The qualitative component explored clinicians’ baseline knowledge, comfort, confidence, current practice, and perceived barriers and facilitators to identifying IPV before and after Sensitive Inquiry training. Data were collected via two focus groups (pre- and post-training, ~ 60 min each) and one interview (~ 20 min) for a participant unable to attend the first group. Sessions were held two weeks before and three months after the intervention to capture changes and allow time for practice implementation. Participants chose focus groups or interviews based on preference and availability, with all data collection conducted in person during work hours to minimise disruption. A structured interview guide (Sect.  1.1, Supplementary Material) was used for both interviews and focus groups, covering topics such as baseline knowledge of IPV identification and support, and reflections on the training and its impact on confidence. Sessions were facilitated by authors AT and CW, both familiar with the study’s clinical context. Their expertise and contextual knowledge enhanced participant engagement and enriched the discussions.

## Data analysis

### Quantitative analysis

Inferential analysis used beta regression to examine changes in IPV detection rates before and after Sensitive Inquiry training. The primary outcome was the proportion of patients diagnosed with IPV-related trauma, identified from ICD-10 coding of routinely recorded clinical data. Detection was coded as a binary variable (1 = IPV detected, 0 = not detected) for each patient encounter, enabling modelling of detected cases as a proportion of eligible encounters.

Detection rates were compared across two periods: 12 months pre- and post-training. Models controlled for gender, study week, and COVID-19. Two models were fitted: (1) a pre-COVID model including data up to the first lockdown (March 1, 2020), with study week, intervention, gender, time since intervention, and their interaction as fixed effects; and (2) a post-COVID model focusing on post-intervention data after the state-wide lockdown (week 54), with COVID and its interaction with study week included. A four-week washout period followed intervention implementation. Quantitative analyses were conducted using R (4.2.1) software.

### Qualitative analysis

Qualitative data were analysed thematically using an approach that began inductively and was then mapped deductively to the Theoretical Domains Framework (TDF) [38]. Three researchers (AT, CW, SC) first examined the data to gain familiarity, followed by organisation and inductive manual coding of this data in Microsoft Excel [39] with support from SMM. Codes were reviewed, refined, and organised into themes, which aligned closely with the TDF. Given this fit, findings and quotes were deductively mapped to the framework to facilitate systematic pre–post comparisons, enhance generalisability, and identify targets for future implementation strategies. Two additional researchers experienced with TDF (BA, SH) assisted with mapping. Provisional findings were shared with participating clinicians for feedback and validation, ensuring themes reflected their experiences. Final results were summarised in a pre–post table with illustrative quotes.

### Mixed method analysis

After completing qualitative and quantitative analysis, a convergent synthesis approach was employed to integrate findings from both analyses. This approach involved considering the findings from both datasets to identify areas of convergence and divergence. Any discord among the results was followed by returning to the data for further consideration. This process was facilitated by joint discussions among the research team, ensuring that the synthesis was thorough and reflective of the underlying quantitative and qualitative findings.

## Results

### Sociodemographic description of the training workshop participants

Fifteen participants attended the pre-training focus group: 12 physiotherapists (6 male, 6 female; work experience 4 months to 20 + years) and 3 social workers (all female; work experience 3 months to 5 years). Most physiotherapists were currently working in or had rotated through orthopaedic wards, though only three reported prior IPV training, delivered informally by ward-based social workers. Six participants (3 physiotherapists, 3 social workers) returned for the post-training focus group, joined by six new attendees (3 physiotherapists, 3 allied health students). Attendance variation reflected the physiotherapy department’s routine staff rotation policy across clinical units within the hospital.

### Quantitative results

In total, 12,335 unique patients met eligibility criteria, with a mean age of 36.7 years (SD = 12.6); 38.6% (*n* = 5,652) were female. The mean age of females (36.4, SD = 12.8) was similar to males (36.8, SD = 12.5). Across the study period there were 14,653 encounters, with the 20 most frequent ICD-10 diagnoses presented in Table S1. The most common were adverse effects of benzodiazepines (*n* = 418, 2.9%), unspecified head injuries (*n* = 301, 2.1%), and brief loss of consciousness (*n* = 240, 1.6%). Of all encounters, 927 (6.3%) were IPV-related, most of which involved females (*n* = 665, 71.7%).

**Fig. 2:**
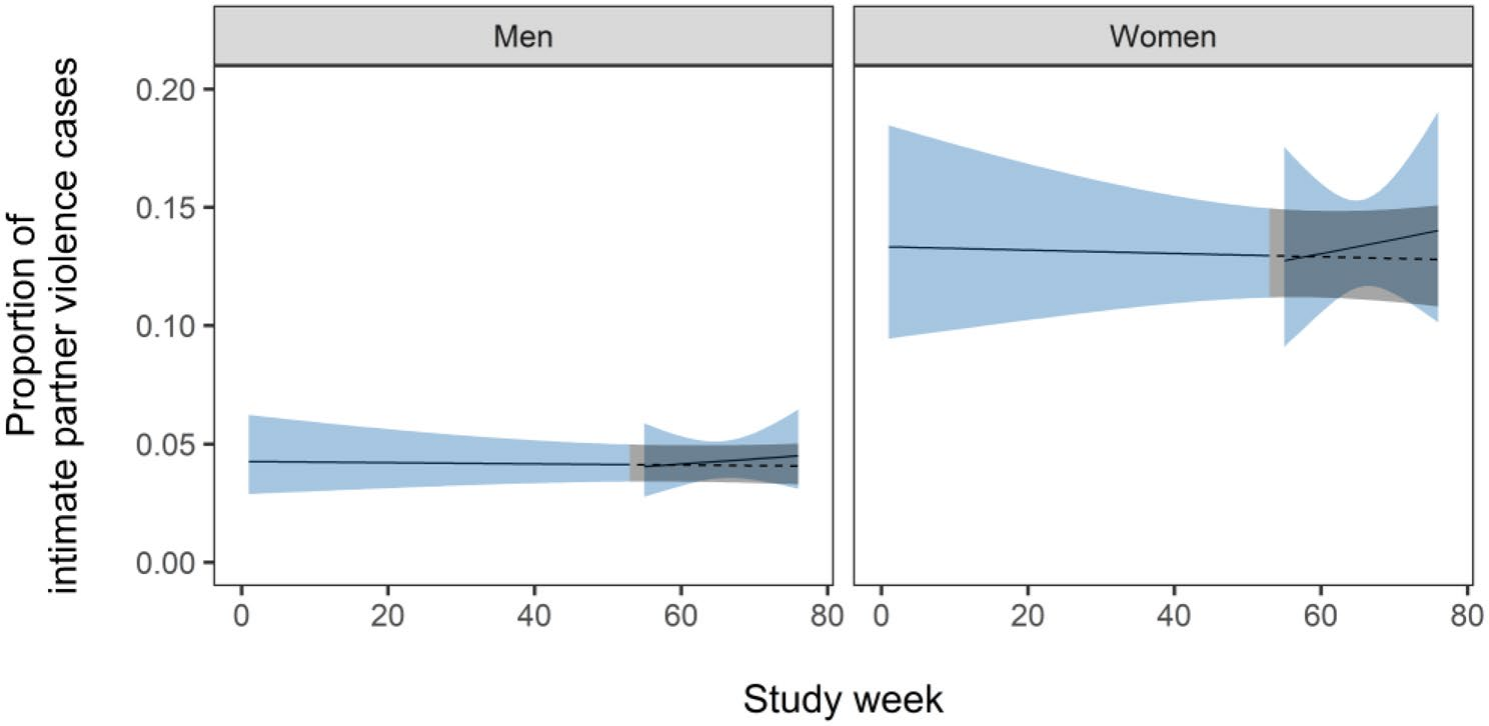
The proportion of intimate partner violence cases over 77 weeks of the study, until the COVID-19 lockdown in March 2020. The solid line indicates the mean proportion and the blue shaded region the 95% confidence interval. The dashed line and grey shaded region indicate the trajectory of expected cases in the absence of the intervention. A higher proportion represents improved detection of intimate partner violence

**Fig. 3:**
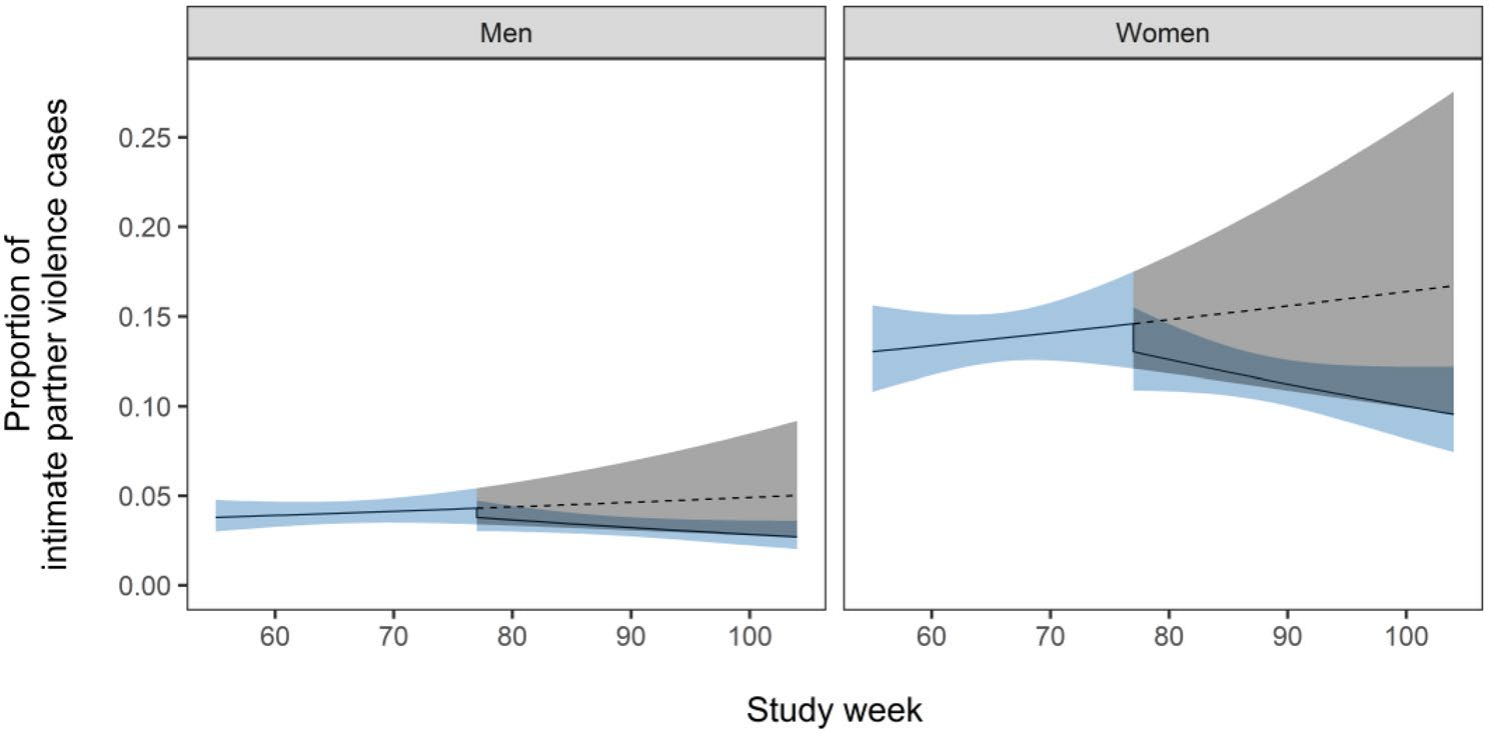
The proportion of intimate partner violence for 12-months after the sensitive inquiry training was introduced. The solid line indicates the mean proportion and the blue shaded region the 95% confidence interval. The dashed line and grey shaded region indicate the trajectory of expected cases in the absence of the COVID-19 pandemic, with state-wide lockdowns introduced in week 77 of the study

The pre-COVID model indicated the brief education intervention was followed by neither a significant immediate increase in IPV detection nor a change in trajectory (Fig. 2; Table 1). Female patients had substantially higher odds of an IPV-related encounter than males (OR = 3.46, 95% CI = 2.92 to 4.10). The intervention-only model suggested COVID-19 had no statistical effect on IPV detection (Fig. 3; Table 2), though uncertainty around the immediate impact of lockdown was high (OR = 3.74, 95% CI = 0.68 to 20.91). Female patients again had higher odds of experiencing IPV than males.

**Table 1:** Parameter estimates from the model using data before the COVID-19 pandemic

Parameter	Estimate	SE	t-value	95% CI lower	95% CI upper
**Intercept**	**–3.210**	**0.119**	**–26.967**	**–3.444**	**–2.975**
Study week	–0.001	0.003	–0.205	–0.007	0.005
Intervention	–0.339	0.958	–0.354	–2.229	1.550
**Gender—Female**	**1.240**	**0.084**	**14.708**	**1.073**	**1.406**
Time intervention	0.009	0.012	0.771	–0.015	0.033
Study week by Intervention	0.006	0.015	0.393	–0.024	0.035

Note. Boldface indicates a statistically important effect. CI = Confidence interval, SE = Standard error

**Table 2:** Parameter estimates from the model including data during the COVID-19 pandemic

Parameter	Estimate	SE	t-value	95% CI lower	95% CI upper
**Intercept**	**–3.562**	**0.556**	**–6.405**	**–4.660**	**–2.464**
Study week	0.006	0.008	0.715	–0.011	0.022
COVID-19	1.324	0.868	1.526	–0.390	3.037
**Gender—Female**	**1.334**	**0.086**	**15.441**	**1.163**	**1.505**
Study week by COVID-19	–0.019	0.011	–1.674	–0.041	0.003

Note. Boldface indicates a statistically important effect. CI = Confidence interval, SE = Standard error

### Qualitative results

Table 3  presents the qualitative findings from pre- and post-education interviews, mapped to the Theoretical Domains Framework with illustrative quotes and key changes. Before training, participants reported limited knowledge, skills, and confidence in detecting IPV, describing discomfort in raising the issue with patients. Some felt it was not their responsibility and preferred to defer to others perceived as more qualified.

*I would say I would not feel comfortable or confident either but … I would be able to do it. I think it’s harder potentially in a ward environment and in that situation*,* maybe it would be more likely to seek out other professionals such as yourselves (gestured to moderators).* (S6)

Pre-training reflections also identified environmental contexts, including time constraints, language, structural limitations and ward culture as barriers to opportunities for sensitive conversations. One participant noted that:

*the language and the attitude (of clinical teams) that surrounds the patient is ‘get you better and off you go’*,* which can act as a barrier to building rapport*,* engaging in sensitive inquiry*,* and providing interventions where consent is given. (S16)*

Feelings of powerlessness were also noted, with systemic barriers such as lack of private spaces and the presence of perpetrators during treatment cited as constraints. After the education sessions, participants described clear gains in knowledge, skills, and confidence, with a more positive outlook and greater recognition of opportunities for engagement.

*I still think that overall*,* as a physio team*,* there’s been a lot more ability to identify those sorts of behaviours or red flags that might be domestic and family violence. And a lot more discussions. (S17)*

Some reported detecting IPV using Sensitive Inquiry and expressed feeling more empowered to take responsibility, while still acknowledging ongoing individual and systemic challenges. These positive shifts in attitudes and perceived roles were consistent across disciplines, and for some participants, sustained and transferred beyond the initial clinical setting.

*I know that I have moved out of Ortho*,* I’m still doing the sensitive line of inquiry in the different areas. I am still using that skill that I learnt. I would probably say five – six since the last focus group that I have identified*,* or people have mentioned. (S15)*

**Table 3:** Summary of qualitative findings mapped to Theoretical Domains Framework (TDF) with illustrative pre- and post-training quotes

TDF domains and definitions	Brief summary of changes noted after the training program	Sample pre-training quotes	Sample post-training quotes
KNOWLEDGE An awareness of the existence of something	Pre-training reflections showed a general awareness of traditional IPV risk factors such as pregnancy, social isolation, and substance use. However, post-training insights revealed an expanded understanding that risk is not limited to specific groups and can apply more broadly. Participants began to view IPV as a concern relevant across diverse patient contexts, reflecting a shift toward a more inclusive and nuanced understanding of vulnerability.	“As S3 was saying before with the isolation, that’s one of the risk factors, I know the risk goes up during pregnancy, if there’s things like financial dependence upon a partner. So, anything that disempowers the victim I think increases the risk of domestic and family violence. And then other things like drug and alcohol abuse within the family unit, whether that’s the perpetrator or the victim.” (S7) “I think there’s an increased risk of violence all the time when the victim tries to leave the relationship attempts tried to leave a domestic violence situation.” (S8)	“I remember after the first training session, you sort of go back into the ward and you’re looking at every visitor differently, thinking…you almost go overboard thinking that almost everyone coming through that door, you know ‘what’s going on?” (S3)
SKILLS An ability or proficiency acquired through practice	Before the training, participants described minimal exposure to intimate partner violence identification and limited direct inquiry into patients’ relationships. Post-training, some staff began applying the sensitive line of inquiry, citing real examples of implementation.	“In my four and a half years working here at the (hospital name), I have had very minimal exposure, so I’m not really identifying and responding at all.” (S7) “Look, I don’t think I’ve ever been the first as it’s always been documented prior either in Emergency Department or one of the nursing staff may have brought attention and got a Social Work review….” (S10)	“We had an OT two weeks ago who implemented sensitive line of inquiry after a bit of discussion with S17 about a patient she was concerned about. It has absolutely made a difference.” (S10)
SOCIAL/PROFESSIONAL ROLE AND IDENTITY A coherent set of behaviours and displayed personal qualities of an individual in a social or work setting	Pre-training, some participants already recognized intimate partner violence response as part of their professional role. Post-training reflections, however, indicated a deeper appreciation for the relational nuances of initiating inquiry. Participants discussed the importance of rapport-building and tailoring their approach based on patient readiness, signalling a shift toward more patient-centred, reflective practice.	“…… I feel it is my responsibility to do so (responding to intimate partner violence), and to create an environment where I can ask that question” (S9) “I think I see a lot of inequality on the orthopaedics ward in how nurses respond to victims of domestic violence if they’re drug users. So, I think that’s always a challenge in delivering the same amount of empathy and compassion to these patients. As someone who is a professional woman that’s experienced domestic violence and everyone suddenly feels really sorry for, I think that’s a challenge.” (S1)	“I think, just reflecting on it, there’s going to be a fine line and a fine balance to make between building enough rapport that they can feel like they can disclose that information rather than just going in …. that’s how I approached it……. that might be too early for some patients to ask that question so you might need to build that rapport. And I guess what I’m trying to say is that I think the disclosure is going to change based on that interaction and the rapport you build with them.” (S19)
BELIEFS ABOUT CAPABILITIES Acceptance of the truth, reality, or validity about an ability, talent, or facility that a person can put to constructive use	While pre-training quotes highlighted a lack of confidence and discomfort with intimate partner violence inquiry, post-training responses showed greater perceived ability to apply questioning strategies, access resources, and respond more appropriately. Though some discomfort remained, there was evidence of increased self-efficacy and a sense of preparedness in real clinical interactions.	“I think I don’t feel confident at all, but I do feel the longer you get to speak with someone who is in a situation like this, the more likely you are to have an understanding that everything is not as you’d expect or not as you assume.” (S3) “No, I don’t feel comfortable or confident, but when I’ve seen a patient where I’m suspicious of it… I don’t like doing it, but I feel it is my responsibility to create an environment where I can ask that question” (S9)	“I think with the training that we’ve undertaken of the basic understanding of the line of questioning and the way to go from a questioning perspective, and then I feel like I’ve got a pretty good idea of the resources that I’ve got to help me to respond appropriately to that line of questioning.” (S3) “I didn’t go in thinking I would ask the question. When I went in, she provided a little bit of that insight when I asked about her home situation, what supports she had at home if she was to discharge. And then I thought ok this might be a good opportunity to probe a bit further about what she had at home and that’s when. My line of thinking was that I would ask her if she felt safe at home. Kinda what we talked about at our last in-service.” (S19)
OPTIMISM The confidence that things will happen for the best or that desired goals will be attained	Although no pre-training quotes were captured for this domain, post-training reflections suggested growing optimism about team-wide improvement. Participants noted more frequent discussions among colleagues and increased identification of potential red flags, indicating a more hopeful outlook on their collective ability to address intimate partner violence in practice.	Not captured.	“I think what’s really shown to me from the whole team is there’s been more conversations with all of us and more, ‘hey I just want to run this by you’? So, I think everyone’s actually become a lot better at identifying the signs and whether they are comfortable or not approaching the patient or using the words…… which has been really great for us.” (S17) “I think that it stirs buzz with nursing staff too, which is always helpful when we’re not around all the time or we are not involved in the patient” (S16)
BELIEFS ABOUT CONSEQUENCES Acceptance of the truth, reality, or validity about outcomes of a behaviour in a given situation	Participants articulated an increased awareness of how intimate partner violence can impact physical and mental health, discharge decisions, and recovery. Post-training reflections included more nuanced insights into how Sensitive Inquiry can directly affect patient outcomes, such as improved readiness for discharge and empowerment. This suggests a shift toward recognising the broader consequences of unaddressed intimate partner violence and the value of trauma-informed care.	“Kind of hard to imagine- think of yourself having an orthopaedic injury and then going home to a supportive welcoming environment and you think of these people not going back to that environment at all…so the mind boggles as to the impact that has on both physical and mental health outcomes.” (S3)	“the first time I saw her, her mobility and confidence were really quite low. And I think she was probably thinking of the impact that that would have at home. I guess the sensitive line of inquiry helped me to identify that that was probably one of the contributing barriers, but that the next day, with her confidence up and with her mobility up she was a changed person. And, you know, she was very much talking about going home today. I guess in the past, I might not have been asking enough questions to figure out why the barrier was there and probably would have just pushed a little harder to make that discharge happen the day before.” (S3)
REINFORCEMENT Increasing the probability of a response by arranging a dependent relationship, or contingency, between the response and a given stimulus	Although no pre-training data were available for this domain, post-training reflections emphasised the value of environmental prompts such as visible reminders and regular in-service discussions to reinforce Sensitive Inquiry practices. This may indicate a developing awareness of how cues and social reinforcement can help normalise and sustain the behaviour over time.	Not captured.	“I think having that piece of paper to remind us in the office. Next step could be templates in the chart…. It might prompt you to remember. Things like that would probably make it routine practice. And then regular in-services.” (S19) “I agree, just having more conversations in the office about this type of thing. Checking in, how is the sensitive line of inquiry going? That’s the way to go. And I think we’re starting to do that a bit more.” (S5)
INTENTIONS A conscious decision to perform a behaviour or a resolve to act in a certain way	Prior to training, some clinicians expressed reluctance or noted that they delegated the task of intimate partner violence inquiry. After training, participants described a clearer intention to engage in sensitive inquiry themselves, with some reporting multiple cases identified since the intervention. There was also evidence of increased consideration of relationship safety in routine assessments, suggesting a shift from avoidance to deliberate action.	“Um, I’m going to say no on that one because it’s (intimate partner violence inquiry) probably something I ask other staff to do and not do myself. I’m comfortable in acknowledging that there is a risk and assessing that, but then I probably palm that task off onto somebody else.” (S1)	“I feel like I am thinking about it in my head a bit more. I haven’t actually gone down specifically asking a patient the sensitive line of inquiry but definitely has raised my awareness to bring it up or that there might be a potential for violence at home at the board rounds and with the MDT (multi-disciplinary team). That is some of the benefits that we have seen.” (S5)
GOALS Mental representations of outcomes or end states that an individual wants to achieve	While pre-training data were not available, post-training reflections revealed an emerging goal orientation, with clinicians expressing a desire to expand training and support beyond their own roles to include nursing and broader multidisciplinary teams. This reflects a growing commitment to system-level change and sustainability of Sensitive Inquiry practices.	Not captured.	“It’s certainly a will to continue. I wonder if this has been a physio/social work specific project, but I think our next step that we need to look at is how do we expand this out to the other members of the orthopaedic team? Nursing being 24/7 is going to be a real asset moving forward and, you know, other members of allied health team as well. How do we some education and some training and make sure the whole team is involved.” (S5)
MEMORY, ATTENTION AND DECISION PROCESSES The ability to retain information, focus selectively on aspects of the environment and choose between two or more alternatives	Post-training reflections indicated that remembering to ask about IPV remained a challenge for some clinicians. Participants suggested practical solutions such as visible cue sheets and prompts within the workspace to help build automaticity. This highlights a shift toward using cognitive supports to embed sensitive inquiry into routine decision-making.	Not captured.	“On of the barriers is just remembering sometimes, I think part of that in-service, …. you had a sheet up in our office with questions that you start the conversation with. …………… Until it becomes second nature. that were minor things to overcome the barrier of just remembering until it becomes second nature.” (S19)
ENVIRONMENTAL CONTEXT AND RESOURCES Any circumstance of a persons’ situation or environment that discourages or encourages the development of skills and abilities, independence, social competence, and adaptive behaviour	Pre- and post-training reflections consistently highlighted environmental barriers such as lack of privacy, time constraints, and ward culture. However, post-training insights revealed a growing awareness of how these contextual factors influence inquiry practices. Some participants expressed increased motivation to navigate these challenges, for example, by shifting the mindset that IPV inquiry is not part of their role and by adjusting their workflow to create opportunities for sensitive conversations when possible.	“I guess one of the barriers would be if they are always accompanied by their partner or family member or whatever and you didn’t feel you could ask the question while they were there.” (S8) “I don’t have a lot of violence in my life or in the people around me so… you think of everyone having this lovely supportive environment that I’ve got to live in…. I guess I’m just not wired to be thinking along those lines.” (S14)	“…. I have never seen it (intimate partner violence inquiry) as my job, as a young clinician I wouldn’t have gone anywhere near it. As an older one I feel more saying ‘oh that was a bit ridiculous. I think this is a little bit in that zone for me, and I’m trying to get it out of that zone because of the seriousness and how prevalent it is. But I do find it hard.” (S10) “…. I think from a whole orthopaedic unit perspective, we’re no longer having discussions around why doesn’t she just leave? I think as a unit we matured beyond that, I think whether that’s the role that you guys have had over the years from a social worker perspective or whether that’s overall, as a facility, we’re increasing our awareness. I think, you know, we can move from that point to now understanding the complexities.” (S3)
SOCIAL INFLUENCES Those interpersonal processes that can cause individuals to change their thoughts, feelings, or behaviours	Participants described increased support from colleagues and a shared sense of responsibility after the training. While pre-training reflections focused on structural limitations (e.g., needing a policy to remove partners from the room), post-training data showed enhanced peer support, more frequent team discussions, and a willingness to challenge problematic attitudes. This suggests that social norms and team dynamics around intimate partner violence inquiry had begun to shift.	“I think in the ED [emergency department] setting it to be almost like there is a policy of that there is a period of time which we could fall back on, whereby it’s the clinician and the patient only with no family members. So, you can feel free to begin that line of inquiry. Sometimes it’s really hard to come up with the reason why the partner can’t be in the room.…. it would be nice to have a policy that you can point to and say, hey look this is hospital policy so you can start that sensitive inquiry.” (S9)	“I think it’s probably got me thinking a little bit more about some of the behaviour that could lead into this sort of stuff. So, patient in my room the other day talking about wanting to go for a walk out to the corridor so he could check out the chickee babes out there, you know, pulling him up on that. Just the noting that the nurses on our ward are fabulous and do a great job and it’s a bit disrespectful to call them chickees. I think, you know, maybe that’s not domestic and family violence, but we’re talking about behaviours here. People need to be pulled up on it and spoken about it a bit more.” (S3) “I definitely felt with some, actually with all of them when we’ve been able to identify and work as a team, the support we as social work get from the team, including some of the doctors and nurses now we are not rushing people out the door compared to when I started here two years ago in orthopaedics. It is remarkably different…. It wasn’t anything significant, but it was enough, with the few cases I’m thinking of, to build the confidence of the patients to go the next day instead of today. It has been a high difference to have that support.” (S16)
EMOTIONS A complex reaction pattern, involving experiential, behavioural, and physiological elements, by which the individual attempts to deal with a personal significant matter or event	Pre-training comments indicated discomfort, awkwardness, and emotional hesitation when approaching the topic of intimate partner violence. Post-training reflections revealed that while some emotional discomfort remained, participants were becoming more reflective and self-aware about these reactions. Several expressed that as sensitive line of inquiry becomes more embedded in everyday practice, emotional barriers would likely diminish.	“You are likely going to be sending the person back to that area where they have had that harm done or that environment. So, in the back of my mind, it’s like, it’s just like I’m going to be sending them straight back from where they came from. We can’t get them the help there and then in that situation again.” (S14)	“I think for me it’s probably not a normal part of my practice in terms of questioning at the moment. So, I think I had to think in my head how I go about asking further. I guess it’s just a bit unnatural at the moment. …. and the fact that she then became probably a bit too emotional for me to continue on with anything else I was doing.” (S19) “I had one particular patient to talk to me about the amount of alcohol that is consumed by her partner just over the weekend. We talked about that and what normal levels were. And then when I walked away, I thought that was my opening to ask, but then I didn’t feel like I could come back to that. That was my own feeling of being uncomfortable with the situation. I suppose I need a bit longer to get comfortable about asking it for multiple reasons.” (S10)
BEHAVIOURAL REGULATION Anything aimed at managing or changing objectively observed or measured actions	Post-training data emphasised the importance of regular reinforcement and ongoing training to maintain skills and confidence. Participants recognized that one-off sessions were insufficient and began advocating for repeat in-services and reflective opportunities. This demonstrates increased awareness of what is needed to support lasting behaviour change in clinical settings.	Not captured.	“It does really feel like it’s the sort of one off or two off training isn’t going to cut it. I guess now we have a couple of us that has had this little experience that we’ve had and so it’s like, oh, we can build on that. And if someone hasn’t had, it means that maybe they can learn from the experiences of the individuals. And then in a month’s time, it’s the next little input. It seems like the thing you need a stack of training, ongoing training to one, get the skills and two, maintain those skills. Because it is so uncomfortable.” (S3)

### Integration of quantitative and qualitative findings

Integration of quantitative and qualitative findings revealed divergence in the observed effects of the brief education intervention. Quantitative analyses did not demonstrate a statistically significant immediate or sustained increase in IPV detection following the intervention, either before or during the COVID-19 period. In contrast, qualitative findings indicated perceived improvements in participants’ knowledge, skills, confidence, and sense of responsibility regarding Sensitive Inquiry for IPV. Post-training interviews suggested increased readiness to initiate conversations about IPV and greater recognition of opportunities for engagement across disciplines. Together, these findings suggest that although the intervention demonstrated positive changes in clinician capability and attitudes, these did not translate into measurable changes in IPV detection at the service level within the study period.

## Discussion

Although clinician participants who completed the brief education intervention reported positive perceptions of using Sensitive Inquiry to support the detection and referral of people experiencing IPV, there was no observed change in detection rates at the participating hospital among patients presenting with diagnoses commonly associated with such violence. When considering the distinct contributions of each data source, the findings suggest that this brief educational intervention alone was insufficient to produce a sustained, meaningful increase in the detection of IPV. This may be attributed, in part, to the limited scale of the intervention, both in terms of the number of staff reached and the high turnover among clinical personnel. Additionally, it may be the case that the one-year post-intervention period was too short to assess the quantitative impact of the training program as IPV survivors often exhibit nonlinear response patterns with delayed disclosure and engagement [40]. Moreover, changing clinical outcomes (detection rates) in this study was dependent first on achieving individual healthcare provider change which can often be a slow and complex process [41]. However, the improvements in provider knowledge and confidence observed in our qualitative analysis represent foundational precursors to such behaviour change. While changes in individual behaviours such as implementing Sensitive Inquiry in practice, identifying IPV survivors or providing support services were not quantitatively measured in our study, they were reflected in qualitative accounts post-training, suggesting potential effectiveness of the training on individual behaviour change. Consequently, planning medium- and long-term evaluations with a broader range of outcome measures to capture changes that materialise over time will be important [42].

These results also indicate that more intensive, multi-component interventions may be required to produce demonstrable changes in IPV detection rates. Such approaches could include regular, ongoing training; embedded decision-support systems for clinicians; and longer-term implementation strategies to overcome the systemic barriers to IPV detection within complex hospital settings. Participants frequently cited challenges such as the lack of opportunities for post-training reinforcement, inadequate physical environments for conducting private conversations, and insufficient workforce capacity to allow time for sensitive engagement with patients. These challenges also included factors related to language and attitudes of teams within the hospital environmental context.

Addressing these challenges is likely to require strong organizational leadership to support sustained implementation, foster cultural change, and ensure alignment with institutional policies, procedures, and clinical guidelines, particularly those informed by frameworks such as the *Strengthening Hospital Responses to Family Violence* model [29].

The lack of significant change in the rate of IPV detection in our study was an interesting finding. There are several possible explanations for this non-significant finding. The Sensitive Inquiry training in the current study was brief, which may have limited its effectiveness. Successful training programs are typically longer, and consist of several training sessions [28]. However, the present study was limited in scope due to constrained resources, including workforce availability in the hospital environment which was further complicated by the COVID-19 pandemic. Additionally, this impact of the pandemic on the broader context in which the training was implemented at the time of the study may have also influenced its effectiveness. Shortly after the Sensitive Inquiry training was introduced, the region where the study was conducted was placed in a government mandated lockdown, which restricted people’s movement in the community to reduce risk of virus transmission. During this period, increased rates of alcohol consumption were reported in the community [43] and there was general concern within the community about the potential for increased coercive control by perpetrators [44]. It is possible that increases in coercive control reduced the ability of IPV survivors to seek help at this time [44]. It is also possible that people were avoiding healthcare, and particularly hospitals, due to a perceived risk of contracting the SARS-CoV-2 virus [45].

It is also plausible that the use of routine medical record coding was an inaccurate representation of change in detection rate, although this is perhaps less likely than the former possible explanations as each medical record is manually reviewed by skilled hospital coders after each encounter. Nonetheless, IPV can only be coded if it is clearly and appropriately recorded within the medical record. We therefore concluded that the lack of significant change in detected IPV-related encounters following the Sensitive Inquiry training intervention was likely attributable to a combination of factors: the absence of a sustained effect from brief training alone, lag between implementation and effect on longer term outcomes, potential hospital avoidance (deliberate or otherwise), and routine documentation practices that may not have accurately reflected actual detection rates.

Despite the non-significant findings of the standalone brief education approach in the present study, more effective and sustained implementation of specialised IPV training is likely to be a critical component of multi-component interventions to increase detection rates by health services. Institutional endorsement of curricular content, facilitator expertise and funding availability have all been reported as facilitators to implementation [46]. Previous studies have identified professional uncertainty, the maintenance of nonjudgmental attitudes, management of own feelings and personal domestic violence experiences [47] and stigma [48] as important screening enablers. Service related barriers have included high patient throughput, an absence of electronic prompts and a lack of privacy in the clinical setting [49].

While traditional screening methods alone appear ineffective in detecting IPV [50, 51], collaborative service models have shown promise [47]. However, care coordination, including with community agencies that may be referral targets, have remained a major challenge [47]. Nonetheless, coordinated care will likely be an important factor in overcoming barriers to improved IPV detection and care [52] in the context of future multi-level implementation strategies. We found that IPV-related encounters accounted for 6.3% of all traumatic-injury encounters across the study period. This could suggest that 2006 Australian estimates of 2.4 to 3.4% [53]. are a gross underestimation and this study adds weight to prior work highlighting the importance of effectively addressing the issue of IPV.

Several limitations affect the interpretation and generalisability of this study. Both the design and intervention were pragmatic to ensure feasibility within a busy metropolitan hospital context further constrained by COVID-19 restrictions. Quantitative outcomes relied on routinely coded hospital records, which, while standardised and robust, may benefit from future validation in the context of IPV detection or the use of bespoke data collection methods. Findings are also limited to a single large metropolitan hospital in a high-income setting and may not extend to different contexts. Finally, although interviews and focus groups were conducted independently, reducing cross-method bias, this design limited the ability to directly link qualitative insights with quantitative changes in IPV detection at the clinician or unit level.

## Conclusion

Intimate partner violence is a serious public health and human rights issue, highlighting the urgent need to strengthen current detection models. In this study, brief Sensitive Inquiry training alone did not increase IPV detection rates. This lack of change may reflect the limited training duration, as well as pandemic-related factors such as reduced healthcare attendance. However, qualitative findings were encouraging, suggesting Sensitive Inquiry may play an important role when embedded within more intensive, multi-faceted interventions. Future studies should test regular training as part of multi-component implementation strategies, ideally involving entire multi-disciplinary teams, and evaluate their effectiveness through implementation–effectiveness hybrid trials in Australian healthcare settings.

## Supplementary Information

Below is the link to the electronic supplementary material.

Supplementary Material 1

## Data Availability

Data is provided within the manuscript or supplementary information files. There are no additional data that are shareable within current ethics and governance approvals for this study.

## Declarations

### Supplementary Information

GRAMMS and TIDieR checklists; Interview guides used for pre- and post-training focus group discussions and interviews; Table S3. Top 20 most prevalent principal diagnoses from 14,653 encounters; Table S4. Intimate partner violence-related ICD-10-AMa codes and associated descriptions.

## Abbreviations

WHO: World Health Organization
IPV: Intimate partner violence
COVID-19: Coronavirus disease (SARS-CoV-2 virus)
GRAMMS: Good Reporting of A Mixed Methods Study
TIDieR: Template for Intervention Description and Replication
HREC: Metro South Hospital and Health Service Human Research Ethics Committee
ICD-10: International Statistical Classification of Diseases and Related Health Problems, 10th Revision
DFV: Domestic and Family Violence
TDF: Theoretical Domains Framework
