# Supplementary material for: A brief educational intervention to improve health service responsiveness to intimate partner violence: a mixed methods evaluation
Source: BMC Health Serv Res. 2026 Mar 11;26:542. doi: 10.1186/s12913-026-14277-9 (PMC13088842; doi:10.1186/s12913-026-14277-9)
Supplement: Supplementary file 1 — Supplementary Material 1 [file 12913_2026_14277_MOESM1_ESM.docx]

Supplementary Material

A brief educational intervention to improve health service responsiveness to intimate partner violence: a mixed methods evaluation

Table s1. GRAMMS checklist (O'Cathain et al., 2008)

|  | Item | Page number |
| --- | --- | --- |
| 1 | Describe the justification for using a mixed methods approach to the research question | Page 5 |
| 2 | Describe the design in terms of the purpose, priority and sequence of methods. | Page 6 |
| 3 | Describe each method in terms of sampling, data collection and analysis. | Pages 7, 9, 10, 11 and 12 |
| 4 | Describe where integration has occurred, how it has occurred and who has participated in it. | Page 12 |
| 5 | Describe any limitation of one method associated with the present of the other method. | Pages 16 and 17 |
| 6 | Describe any insights gained from mixing or integrating methods Discussion. | Pages 14, 15 and 16 |

Table s2. Tideir checklist (Hoffmann et al., 2014)

|  | Item | Location |
| --- | --- | --- |
| 1 | BRIEF NAME: Provide the name or a phrase that describes the intervention. | Page 8 |
| 2 | WHY: Describe any rationale, theory or goal of the elements essential to the intervention. | Page 8 |
| 3 | WHAT:  Materials: Describe any physical or informational materials used in the intervention. | Page 8 |
|  | Procedures: Describe each of the procedures, activities, and/or processes used in the intervention. | Pages 8 and 9 |
| 4 | WHO PROVIDED: For each category of intervention provider, describe their expertise, background, and any specific training given. | Page 8 |
| 5 | HOW: Describe the modes of delivery (e.g., face-to-face, telephone) of the intervention. | Page 8 |
| 6 | WHERE: Describe the type(s) of location(s) where the intervention occurred, including any necessary infrastructure or relevant features. | Page 8 |
| 7 | WHEN and HOW MUCH: Describe the number of times the intervention was delivered and over what period of time. | Page8 |
| 8 | TAILORING: If the intervention was planned to be personalized, titrated, or adapted, describe what, why, when, and how. | Page 9 |
| 9 | MODIFICATIONS: If the intervention was modified during the course of the study, describe the changes (what, why, when, and how). | No modifications were done during the study period. |
| 10 | HOW WELL:  Planned: Describe how intervention adherence or fidelity was assessed, and any strategies used to maintain or improve fidelity. | Page 9 |
|  | Actual: Describe the extent to which the intervention was delivered as planned. | Not measured |

**Section 1.1 Interview guides used for pre- and post-training focus group discussions and interviews**

Evaluating a new model of care to improve health service responsiveness

to domestic and family violence

Implementation Evaluation - Focus Group Topic Guide

**Objective:**

Identify factors that supported and barriers that impeded the implementation and success of the project, including factors that may be important for scale‐up or adoption in other Hospital and Health Services.

**Participants:**

Focus groups/interviews will be held with the following key groups as applicable:

‐ The implementation team (Project Lead, DV Training Co-ordinator)

‐ Staff participants

Potential participants will be invited to participate by the Project Lead.

Participation will be voluntary with verbal consent obtained prior to participation. Each focus group will be approximately 60 minutes in length, numbers will be small due to specific designation of staff and work area (Orthopaedic Units). Some questions may be asked one-to-one before or after the focus group /interview.

**How to use this guide:**

The questions are based on the Strengthening Hospital Responses to Family Violence Model (SHRFV – Royal Women’s Hospital and Bendigo Health, Victoria) which provides a framework for embedding the practice of identifying and responding to family violence experienced by patients. This model is based on international best practice.

1. Questions for discussion will be selected from the list below (including some asking of questions one-to-one with participants prior to focus group commencement for key questions that pertain to them as an individual).
2. The facilitator will use responses from the pre-training focus group/interview implementation to select areas for in‐depth discussion in advance of training implementation.
3. Should other relevant issues be raised they will be explored in the relevant section, with prompting as required
4. Facilitators will ensure discussion progresses in a timely, yet informative manner.

**Key questions (pre-training about you):**

1. What is your profession?

- Physiotherapy
- Social Work

1. How long have you worked in this profession?

- <1yr
- 1-5 years
- 6-10yrs
- >10 years

1. Have you had any previous training or in-services on the underlying causes of violence against women?

- Yes
- No

**Key questions (Pre-training Baseline Knowledge):**

1. What is your understanding of the definitions of domestic and family violence?
2. What is your understanding of the risk factors for violence against women?
3. What is your understanding of the health impacts of violence against women?

**Key questions (Pre-training Baseline Comfort & Confidence):**

1. Do you feel confident in identifying the clinical risk indicators of violence against women?
2. Do you feel comfortable and confident asking a patient about family violence?
3. Do you feel comfortable and confident responding to a patient disclosure of family violence?
4. Do you feel comfortable and confident in gauging the level of risk for family violence?
5. Do you feel comfortable and confident to provide referrals to a patient experiencing family violence?
6. Would you feel able to document a patient’s experience of violence (including use of hospital alert systems)?

**Key questions (Pre-training Current Practice):**

1. How often do you identify and respond to concerns of violence against women in the hospital?
2. How often do you initiate inquiry (asking) about experiences of violence against women?
3. What do you think would be of most help to you in identifying and responding to victim/survivors of violence against women in the hospital context?
4. Where do you currently refer your concerns of violence against women?
5. What do you see as the main barriers to identifying (asking) and referring victim/survivors of violence against women in the hospital?

- Patient information concerns (e.g. recording in notes, privacy/medico-legal issues etc.)
- Clinical uncertainty
- Perceptions of liability
- Lack of supporting Policy, Procedures and/or Guidelines
- Lack of awareness of the issue
- Environmental constraints (e.g. lack of private areas to discuss sensitive issues)
- Other (please specify)

**Key questions (Post-training):**

1. Do you think clinicians know how to ask and respond to patients with signs of family violence?
2. Are clinicians are putting sensitive inquiry into practice?
3. What response do you think clinicians receiving – disclosure or non-disclosure?
4. How many patients have made a disclosure of family violence?
5. What type of referrals are being offered – internal or external?
6. Are referrals being accepted?
7. What is the patient perspective of being asked about family violence in the hospital setting?
8. What is the patient perspective of the support / referral provided?
9. Has clinical practice changed as a consequence of the training?
10. Do you have increased capacity and preparedness to facilitate feelings of safety, choice and control during your interactions with women who have experienced IPV after having received the training?
11. How has the training impacted your preparedness to enquire and discuss
12. Are clinicians applying self-care in relation to working with patients who have experienced family violence?
13. Are there any processes in place to ensure sensitive practice becomes routine clinical practice?
    - - Model of clinical champions implements
      - Clinical champions anecdotal reporting

**Table s3. Top 20 most prevalent principal diagnoses from 14,653 encounters.**

| Diagnosis | Description | Number (%) |
| --- | --- | --- |
| T42.4 | Benzodiazepines | 418 (2.85%) |
| S09.9 | Unspecified injury of head | 301 (2.05%) |
| S06.02 | Loss of consciousness of brief duration [<30 min] | 240 (1.64%) |
| T39.1 | 4-Aminophenol derivatives | 240 (1.64%) |
| S19.9 | Unspecified injury of neck | 231 (1.58%) |
| F10.0 | Mental and behavioural disorders due to use of alcohol, acute intoxication | 229 (1.56%) |
| T43.5 | Other and unspecified antipsychotics and neuroleptics | 202 (1.38%) |
| T81.4 | Wound infection following a procedure, not elsewhere classified | 200 (1.36%) |
| S61.0 | Open wound of finger(s) without damage to nail | 175 (1.19%) |
| T81.0 | Haemorrhage and haematoma complicating a procedure, not elsewhere classified | 175 (1.19%) |
| T43.2 | Other and unspecified antidepressants | 169 (1.15%) |
| S06.00 | Concussion | 167 (1.14%) |
| S39.9 | Unspecified injury of abdomen, lower back, and pelvis | 150 (1.02%) |
| T18.1 | Foreign body in oesophagus | 148 (1.01%) |
| S06.5 | Traumatic subdural haemorrhage | 139 (0.95%) |
| S61.88 | Grade II soft tissue damage in open fracture or dislocation of wrist and hand | 136 (0.93%) |
| T40.1 | Poisoning by heroin | 123 (0.84%) |
| S06.01 | Concussion, with open intracranial wound | 119 (0.81%) |
| S13.4 | Sprain of ligaments of cervical spine | 110 (0.75%) |
| S62.63 | Fracture of other finger: Distal phalanx | 110 (2.85%) |

**Table s4. Intimate partner violence-related ICD-10-AM^a^ codes and associated descriptions.**

| Diagnosis | Description |
| --- | --- |
| X85.00 | Assault by drugs, medicaments and biological substances, spouse, or domestic partner |
| X91.00 | Assault by hanging, strangulation and suffocation, spouse, or domestic partner |
| X97.00 | Assault by smoke, fire and flames, spouse, or domestic partner |
| X98.00 | Assault by steam, hot vapours and hot objects, spouse, or domestic partner |
| X99.00 | Assault by knife, spouse, or domestic partner |
| X99.10 | Assault by razor blade, spouse, or domestic partner |
| X99.30 | Assault by glass, spouse, or domestic partner |
| X99.80 | Assault by other specified sharp object, spouse or domestic partner |
| X99.90 | Assault by sharp object, unspecified, spouse or domestic partner |
| Y00.00 | Assault by blunt object, spouse, or domestic partner |
| Y01.00 | Assault by pushing from high place, spouse, or domestic partner |
| Y03.00 | Assault by crashing of car with other motor vehicle, nontraffic, spouse or domestic partner |
| Y03.80 | Assault by other specified crashing of motor vehicle, traffic, spouse, or domestic partner |
| Y04.00 | Assault by bodily force, spouse, or domestic partner |
| Y05.00 | Sexual assault by bodily force, spouse, or domestic partner |
| Y06.00 | Neglect and abandonment, spouse, or domestic partner |
| Y07.00 | Other maltreatment, spouse, or domestic partner |
| Y08.00 | Assault by other specified means, spouse, or domestic partner |
| Y09.00 | Assault by unspecified means, spouse, or domestic partner |
| Z63.0 | Problems in relationship with spouse or partner |

^a^ ICD-10-AM, International Classification of Diseases, 10th Revision, Australian Modification.

**References**

Hoffmann, T. C., Glasziou, P. P., Boutron, I., Milne, R., Perera, R., Moher, D., Altman, D. G., Barbour, V., Macdonald, H., Johnston, M., Lamb, S. E., Dixon-Woods, M., McCulloch, P., Wyatt, J. C., Chan, A. W., & Michie, S. (2014). Better reporting of interventions: template for intervention description and replication (TIDieR) checklist and guide. *Bmj*, *348*, g1687. <https://doi.org/10.1136/bmj.g1687>

O'Cathain, A., Murphy, E., & Nicholl, J. (2008). The quality of mixed methods studies in health services research. *J Health Serv Res Policy*, *13*(2), 92-98. <https://doi.org/10.1258/jhsrp.2007.007074>
